# Augmentation of the Delamination Factor in Drilling of Carbon Fibre-Reinforced Polymer Composites (CFRP)

**DOI:** 10.3390/polym12112461

**Published:** 2020-10-23

**Authors:** Sharizal Ahmad Sobri, David Whitehead, Mazlan Mohamed, Julie Juliewatty Mohamed, Mohd Hazim Mohamad Amini, Andi Hermawan, Mohd Sukhairi Mat Rasat, Azfi Zaidi Mohammad Sofi, Wan Omar Ali Saifuddin Wan Ismail, Mohd Natashah Norizan

**Affiliations:** 1 Advanced Material Research Cluster, Faculty of Bioengineering and Technology, Jeli Campus, Universiti Malaysia Kelantan, Jeli 17600, Malaysia; mazlan.m@umk.edu.my (M.M.); juliewatty.m@umk.edu.my (J.J.M.); hazim.ma@umk.edu.my (M.H.M.A.); andi@umk.edu.my (A.H.); sukhairi@umk.edu.my (M.S.M.R.); azfi.ms@umk.edu.my (A.Z.M.S.); 2 Department of Mechanical, Aerospace and Civil Engineering, The University of Manchester, Sackville Street Building, Sackville Street, Manchester M1 3BB, UK; david.whitehead@manchester.ac.uk; 3 Center of Excellence Geopolymer and Green Technology (CEGeoGTech), Universiti Malaysia Perlis, Kangar 01000, Malaysia; mohdnatashah@unimap.edu.my; 4 Global Entrepreneurship Research and Innovation Centre (GERIC), Kota Campus, Universiti Malaysia Kelantan, Kota Bharu 16100, Malaysia; 5 Faculty of Innovative Design and Technology, Gong Badak Campus, Universiti Sultan Zainal Abidin, Kuala Nerus 21300, Malaysia; woasaifuddin@unisza.edu.my; 6 Faculty of Electronic Engineering Technology, Pauh Putra Campus, Universiti Malaysia Perlis, Arau 02600, Malaysia

**Keywords:** manufacturing operation, drilling, carbon fibre-reinforced polymer (CFRP), delamination factor

## Abstract

Carbon fibre-reinforced polymer (CFRP) composite materials play an increasingly important role in modern manufacturing, and they are among the more prominent materials used in aircraft manufacturing today. However, CFRP is highly prone to delamination and other damage when drilled due to it being extremely strong with a good strength-to-weight ratio and high thermal conductivity. Because of this problem and CFRP’s growing importance in aircraft manufacture, research has focused on the entry and exit holes as indicators of damage occurrence during drilling of screws, rivets, and other types of holes. The inside of the hole was neglected in past research and a proper way to quantify the internal side of a hole by combining the entry and exit hole should be included. To fill this gap and improve the use of CFRP, this paper reports a novel technique to measure the holes by using the extension of the adjusted delamination factor (S_FDSR_) for drilling thick CFRP composites in order to establish the influence of machining input variables on key output measures, i.e., delamination and other damages. The experimental results showed a significant difference in interpretation of the damage during the analysis. Improvement was made by providing better perspectives of identifying hole defects.

## 1. Introduction

By relying on conventional materials, it is impossible to fulfil the requirements of a growing number of applications because the components required are becoming increasingly complex in geometry and shape. Moreover, the size of components is tending towards two extremes, i.e., either to shrink (micro-parts for products such as optical devices and mobile phones) or to considerably grow in size (the Airbus A380 aircraft wing, for instance). Composite materials consist of two or more separate constituents or phases consisting of manufactured or natural materials. The physical or chemical properties of each material are significantly different and separate within the finished/combined structure. Composites are ultimately the most desired form of material in the industry and already obtain widespread adoption in building, furniture, packaging, flooring, panelling and the automotive industry [1,2,3,4]. Moreover, in many high-tech industries, such as aerospace and defence, composites are frequently used [2].

Manufacturing companies around the globe have no choice but to improve the efficiency of their manufacturing processes. Due to competitive market demands, commercial aircraft manufacturers like Airbus and Boeing are now attempting to change the structures of aircrafts to make them lighter and more efficient [5]. In order to achieve this, according to Garrick [5] and Mangalgiri [6], the most important and sought-after aircraft materials today are fibre-reinforced plastics, including carbon fibre-reinforced plasti/polymers (CFRP). A significant proportion of composite materials used in a latest century of significant passenger/commercial aviation; for example, the Airbus A350 and Boeing B787 have a composite content of more than 30% and 50%, respectively [7].

Generally, there is a need for quantitative evaluation of the extent of delamination by observing the damaged areas of composites that result from manufacturing and fabrication processes [8,9,10,11,12,13]. Chen’s concept (F_d_) [14] proposed quantitative analysis through a ratio of the maximum diameter D*_max_*, which is predominately based on the maximum width or diameter of the delamination zone. This evaluation method is the most commonly used by researchers/technologists due to its non-complex mathematical calculation. However, the damage magnitude being measured in this concept is not fully representative of the damage itself. Only the size of the crack and some breakage occurring at the drilling hole entry and exit are quantified directly through visual inspection, i.e., by digital optical microscope. Davim et al. [8] suggested that Chen’s concept should be incorporated with the damage area related to the maximum diameter of the delamination zone. Furthermore, they strongly confirmed that the damage area contribution truly indicates the extent of delamination across the entire rest of the hole periphery. The first part of the F_da_ equation corresponds to Chen’s work (F_d_), which is a conventional delamination factor, and the second part of the equation corresponds the measurement of the damage area proposed by Davim et al. [8]. The motivation behind this proposition, i.e., the extension of adjusted delamination factor (S_FDSR_), is because of the considerable concern about internal damage to the hole caused mostly by the material thickness.

Moreover, in order to support the argument of the thickness concern, Aoyama et al. [15] concluded that the internal damage of the hole was affected by the fibre bundles’ thickness, where the damage increases as the thickness of fibre bundle increases. It did occur at the same position angle. They investigated the effects of drilling printed wiring boards by using small diameter tools, and they believed that delamination occurred along the fibre into the hole surface. Thus, a proper quantitative method of measuring the quality of the internal hole surface is essential to avoid further damage to the hole surface.

The research work of Aoyama et al. [15] identified that the thickness of fibre—including the fibre angle—influenced the quality inside the through hole. As the feed rate increases, the damage increases as the fibre thickness and angle increases. Nonetheless, this phenomenon was described in woven-ply orientation, and the material was glass fibre reinforced polymer (GFRP), which possesses different fibre characteristics compared to CFRP. Moreover, the effects of the cutting mechanism on both machining methods are distinctly different, especially for laser drilling, whereby the back-reflection effect from the glass fibres is an important factor to consider in the machining process. The main idea of modifying the adjusted delamination factor (F_da_) is inspired from one of their findings. They clearly stated that the internal damage increases when the thickness of fibre increases, by using a small diameter drilling for the through hole. This scenario is similar to current research for drilling of thick CFRP, whereby the depth-to-diameter ratio has an effect when drilling thick composites (i.e., when the depth is 3 times higher than the diameter). In addition, the results from mechanical and laser drilling in previous research findings by Sobri et al. [16] also proved that the damage increases when the tool or laser beam is engaged deeper inside the hole. Nardone et al. [17] alternatively developed the materials that adhesively bonded to concrete substrate by implementing mechanically fastened fibre-reinforced polymer systems using steel anchors to secure the laminate to the substrate. The bearing failure is one of the standard modes of failure analysed in this study. Pre-drilling was implemented in the experiments in order to understand the scenario of this failure mode. The degradation of the bearing happens whenever the ratio of the fastener diameter to the strip width is small. It is interesting to note that by increasing the width-to-hole-diameter ratio, one can increase the bearing strength, and this failure mode causes an elongation of the hole. This research highlights that the understanding of the relationship between the fastening and the slip force is crucial if the proposed model is to be applied, and more research is required to resolve this fundamental parameter.

Given all these considerations, the aim of this paper is to present a novel technique to measure the holes by using the extension of adjusted delamination factor (S_FDSR_) for drilling thick CFRP composites in order to establish the influence of machining input variables on key output measures, i.e., delamination and other damages. An experimental design was proposed for drilling thick CFRP composites under distinct cutting conditions. In addition to this, the analysis of the damage by using the latest digital microscope was conducted in order to assess the delamination factor.

## 2. Extension of the Adjusted Delamination Factor (S_FDSR_)

**Figure 1:**
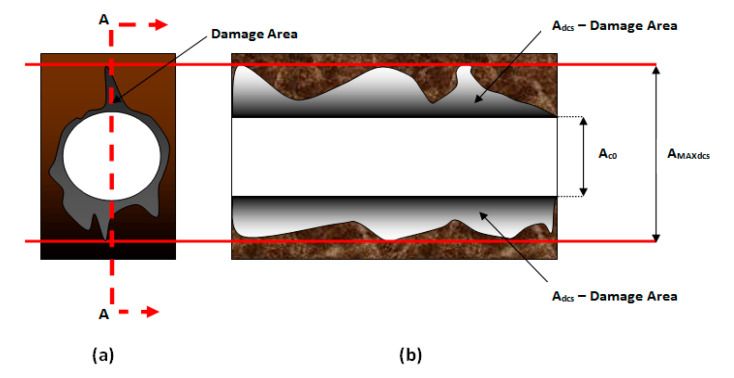
Quantification of the damage area inside the through hole: (**a**) cross-section reference points at the top view of the through hole and (**b**) cross-section view from the point A—A inside the hole.

Characterizing the damage in the delamination zone is a crucial process for researchers or technologists in order to quantify it, and to have a better representation of the damage magnitude for future reference. A novel technique to measure the damage occurs inside the hole or at the cross-section area of the cylindrical hole; the extension of the adjusted delamination factor (S_FDSR_) is introduced and calculated in Equation (1). In Equation (1), the first (i.e., indicated in blue) and second (i.e., indicated in purple) parts represent the concept of F_d_ [14] and the damage area contribution at the hole’s periphery [8], and the third part is the latest element that represents the internal surface area contribution. Figure 1 shows the elements for quantifying the internal surface damage area.

(1) $$S_{FDSR}=\;\alpha \;\frac{D_{max}}{D_{0}}+\;\beta \;\frac{A_{max}}{A_{0}}+2\left(\frac{A_{dcs}}{A_{MAXdcs\;}-\;A_{c0}}\right)$$

Figure 1 provides information of how the cross-section area was measured. It is based on the maximum length of the damaged zone on a cross-section surface. The damage is measured from the point at the edge of an internal hole to the maximum point of damage occurrence. Equation (2) is to compute the maximum length of damage area. This equation calculates the overall damage area, including the area covers on the cross-sectional area of the hole’s cylindrical shape. The function of Equation (3) is to calculate the cross-sectional area of hole’s cylindrical shape. Both equations are applied in the third part of the equation.

(2) $$A_{MAXdcs\;}=Length\;\left(l\right)\;\times \;Width\;\left(w\right)$$

(3) $$A_{c0\;}=\frac{2\pi rh}{2}$$

Substituting Equations (2) and (3) into Equation (1) yields

(4) $$S_{FDSR}=\;\alpha \;\frac{D_{max}}{D_{0}}+\;\beta \;\frac{A_{max}}{A_{0}}+2\left(\frac{A_{dcs}}{\left[Length\;\left(l\right)\;\times \;Width\;\left(w\right)\right]\;-\;\frac{2\pi rh}{2}\;}\right)$$

where the third part of the equation is the ratio of the damage area (*A_dcs_*) to the area corresponding to *A_MAXdcs_* minus the nominal internal surface area or cross-sectional area of the hole’s cylindrical shape (*A_c_*_0_). *A_dcs_* is computed using image digitalisation and processing in order to obtain the damage area. In this particular equation, there is no implementation of weights/parameters like α and β in the first and second parts. This is because these parameters complement each other within the crown area. The crown area embodies the maximum diameter (*D_max_*) of the delamination zone, thus, the internal surface area is not required to include any parameters due to its stand-alone representation of damage magnitude. In other words, the area occurs in a different zone, whereas F_da_ is obtained in the same zone. However, the third part of this equation needs to be multiplied by 2 because the hole’s cylindrical shape is computed for one half; observing the damage in a cross-section view requires the sample to be cut in half in order to examine it. Therefore, the first and second of equation (i.e., based on the work of Davim et al. [8]) combined with the third part can be rewritten as

(5) $$S_{FDSR}=\;\frac{D_{max}}{D_{0}}+\;\frac{A_{d}}{\begin{array}{c} \left(A_{max}-\;A_{0}\right) \end{array}}\;\left(F_{d}^{2}-\;F_{d}\right)+\;2\left(\frac{A_{dcs}}{\left[Length\;\left(l\right)\;\times \;Width\;\left(w\right)\right]\;-\;\frac{2\pi rh}{2}\;}\right)$$

The quantification of internal surface area or cross-sectional area of the hole is able to show a different perspective of the severe damage magnitude. In addition, it is convenient to analyse due to the straightforward mathematical equations, i.e., it is fast and easy to compute and compare the results. Quantification of internal surface area or cross-sectional area of the hole assists researchers or technologists in conducting the analysis by making the results more robust compared to the adjusted delamination factor (F_da_). By doing so, this approach highlights the essential internal structure capability for future reference. This equation is only valid if all holes have reached the same size of the diameter as expected or if the diameter of the hole for both holes is not undersized/oversized by 1 mm from the expected diameter. In addition, the maximum depth of the drilled hole must be reached.

## 3. Materials and Methods

The workpiece material used for the experiments was a multi-directional, multi-layer carbon fibre-reinforced composite with an overall thickness of 25.4 mm (equivalent to 1 inch). The material was supplied in flat panels of 400 (L) × 400 (W) × 25.4 (T) mm, and the panel was cut into blocks of 50 × 20 mm. A visual inspection revealed that the stacking sequence of the lamina was arranged as 0°/90°/−45°/90°/45°/90°/−45°/90°/45°/90°/−45°/90°/0°, and recurred up to 25 m thickness. The thickness of each ply is 0.2 m. The first layer of the CFRP was covered by an additional surface or coating layer that has a thickness of 0.1m. The properties of the CFRP material are shown in Table 1.

A Takisawa MAC-V3 machining centre (Takisawa Machine Tool Co. Ltd., Okayama, Japan) was used to conduct the experiments by drilling an 8 mm diameter hole on each sample in a dry cutting condition. The experimental work used a 2-flute uncoated tungsten carbide (WC) drill bit (WC-uncoated Ø 8 mm with a point angle and helix angle of 118° and 35°, respectively). The selection of the process parameter window as well as the combination of cutting speeds and feed rates were adopted from the work of Sobri et al. [16] (i.e., using a single-step drilling only due to the successful attempt of cutting thick composites). 

**Figure 2:**
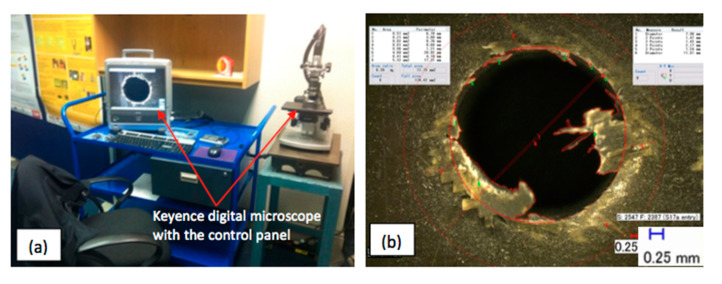
Evaluation of hole quality: (**a**) Keyence digital VHF-500X; (**b**) example of hole entry image analysis.

The one-dimensional delamination factor (F_d_) by Chen [14] is the most commonly applied criterion used by researchers to quantify the amount of damage to a hole produced in fibre-reinforced composite materials during manufacturing processes. Another less often applied criterion is the two-dimensional delamination factor or adjusted delamination factor (F_da_) [8]. This equation quantifies the area of workpiece material where fibres or fibre-bundles have been peeled up or pushed down, blocking off the hole entrance and exit at the nominal hole area. In order to capture the damage that occurred to both the hole entry and exit, a Keyence Digital VHF-500X digital optical microscope (Keyence (UK) Ltd., Milton Keynes, United Kingdom) was used (see Figure 2a). An example is shown in Figure 2b. This microscope was equipped with an image acquisition and measurement system. 

**Figure 3:**
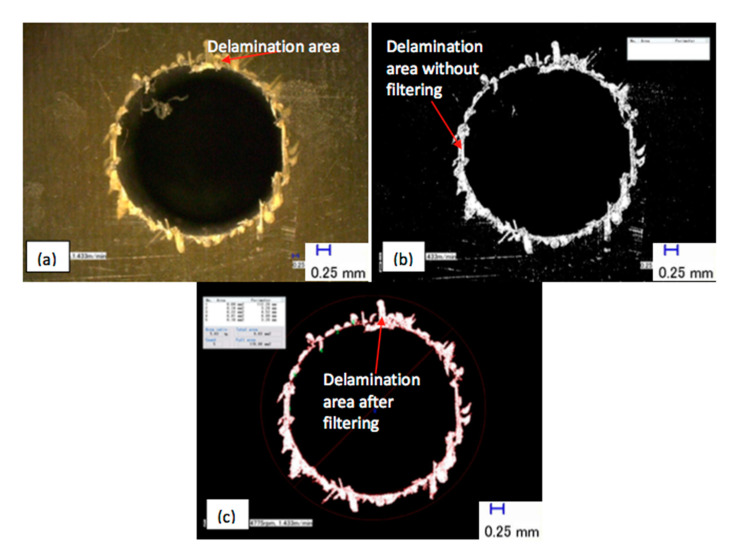
Determination of the damage area by image processing: (**a**) the captured image; (**b**) after initial processing; and (**c**) after the filtering process and the analysis.

Identification of the damage area is a considerable challenge because the visual inspection leads to difficulty in accurately obtaining the exact extent of the damage. In order to aid this procedure, the images obtained with the Keyence microscope were post-processed. By using a discrete process, the software identified the damaged area by pixels that exhibited a higher level of luminous intensity compared to other pixels that belonged to the non-damaged area. It exhibited a lower luminous intensity, as shown in Figure 3. The image then had to be cleaned in order to remove pixels with high luminous intensity that were scattered around the hole. Thereafter, the damage was quantified by manually creating a boundary around the area comprising the pixels of high luminous intensity, both from the inside (i.e., the hole) and the outside (i.e., the surrounding workpiece material). Tests conducted to assess the repeatability of this approach revealed a variation of approximately 10%, which was attributed to the manual definition of the boundary lines. 

**Figure 4:**
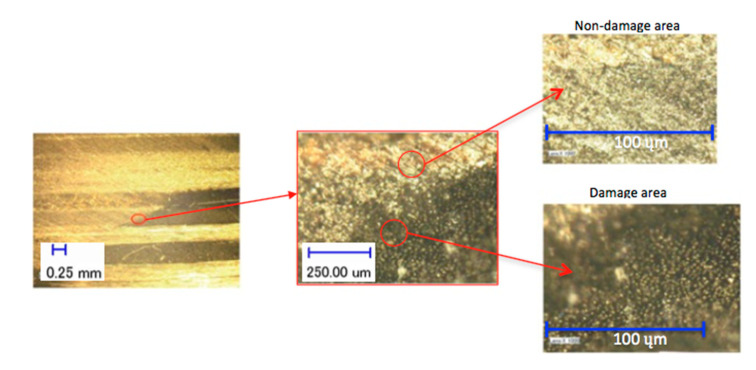
The identification procedure of the damage and non-damage spot/area from laser drilling effects.

The identification of damage along the depth of the hole was conducted in a similar fashion. Figure 4 shows images obtained with the digital microscope taken after the workpiece sample was cut in half along the axis of the laser-drilled hole in order to reveal a cross-section of the CFRP block. The image to the left gives an overview, whereas the image in the centre is a zoomed-in view of the area highlighted by the red circle. This red circle indicates the interface between intact CFRP (top right) and the heat affected zone (bottom right). The heat-affected zone (HAZ) appears as a black region, where the individual carbon fibres can be identified due to the lack or degradation of the surrounding matrix material. The HAZ is measured by quantifying the affected area adjacent to hole periphery, including inside the hole (i.e., cross-section examinations). The average value of the maximum five HAZ length values at the hole periphery and the affected area inside the hole was derived by adding these amounts together (i.e., the five maximum values from the hole periphery and the five maximum values from the affected area inside the hole) and dividing the total by the number of amounts (i.e., 10). 

## 4. Results and Discussion

There was neither a specific guideline nor standard for the hole quality requirement that can be used as a platform to consider the acceptable level of hole quality. The only available information is the results of several pieces of research in the variations of delamination factors in different scenarios. Furthermore, researchers did not develop a standard criterion of which a hole can be considered as being of good quality. In addition, the interpretation of inferior holes was only based on the ratio of delamination factors in both approaches (i.e., F_d_ and F_da_). Researchers mainly considered ratios exceeding 1.200 as severely damaged, resulting in significantly compromised hole quality. Sandvik Coromant [18] has a guideline on hole quality requirements (i.e., a manufacturing company producing drill bits). Based on their guideline, a typical hole quality demand in the aerospace industry is for delamination not exceeding m over the nominal diameter [18], which means the F_d_ ratio should ideally be achieved at 1.125. For example, in the case where the nominal diameter is m and the maximum diameter of the damage zone is m, an F_d_ ratio of 1.125 can be obtained. Nonetheless, this guideline does not specifically relate to material conditions such as thickness, fibre orientation, matrix type, fibre weight ratio, etc. The guideline can also be improvised to satisfy such requirements, since composites of CFRP have different constituents with different fibre arrangements. In addition, the current guideline is mainly applied to the first concept of the delamination factor (F_d_), and it is not applicable for the F_da_ approach, where the ratio value will usually be higher than that for the F_d_ approach.

Based on the discussions in first paragraph, three levels of delamination were standardised in this study to quantify the damage with a better interpretation. These levels of delamination are based on current experimental work, previous findings by other researchers, and the guidelines by Sandvik Coromant [18] as the basis of developing three different levels of damage. The ratio value normally has three decimal places. The first level, indicated from 1.000 to 1.100, shows acceptable hole quality with minimum delamination. Delamination is inevitable when drilling CFRP composites and is almost impossible to avoid. The second, or intermediate, delamination level is represented by 1.101 to 1.200, and can still be acceptable. A post-machining process may be necessary, but a minimum amount of rework is required. Finally, the third and highest level is above 1.201, where the damage is considered to be severe and unacceptable. An amount of rework may be needed, which would incur significant cost if the scenario occurs in mass production. These range values accommodate both F_d_ and F_da_ approaches, including the extension of the adjusted delamination factor (S_FDSR_), and are thus able to provide clear guidance. This guideline can also assist a user or manufacturer to decide which one is considered as having acceptable hole quality with minimum delamination. Thus, all delamination factor results used these three levels to provide essential information about drilling thick CFRP composites.

**Figure 5:**
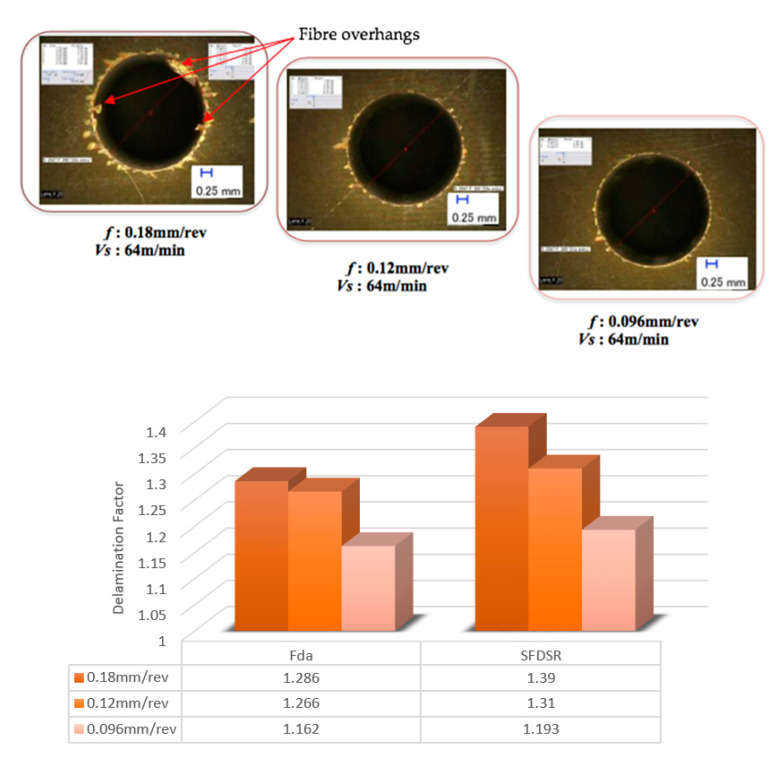
Delamination factor results when drilling at a constant cutting speed of 6 m/min.

**Figure 6:**
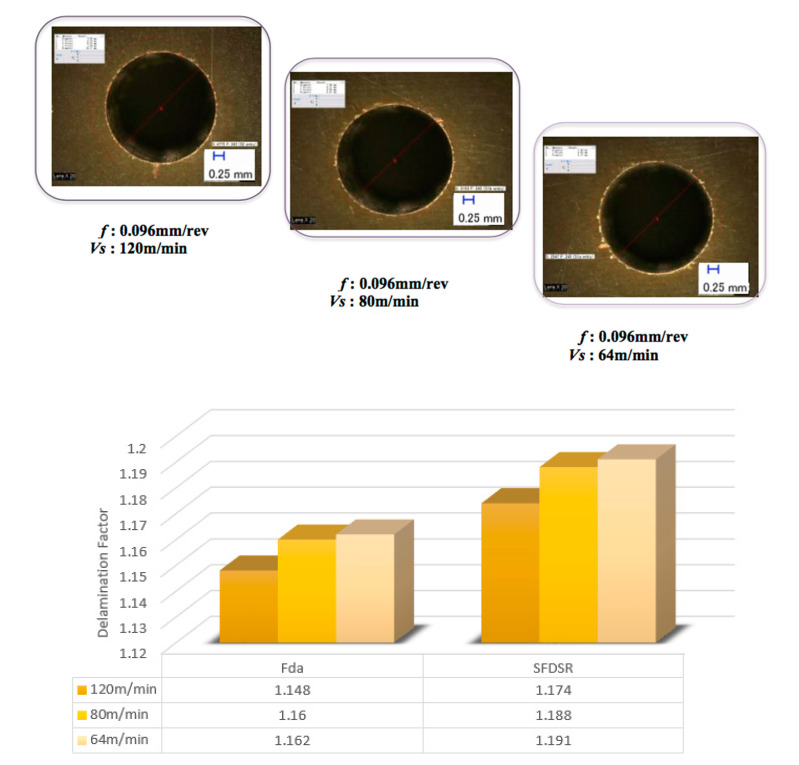
Delamination factor results when drilling at a constant feed rate of 0.09 m/rev.

**Figure 7:**
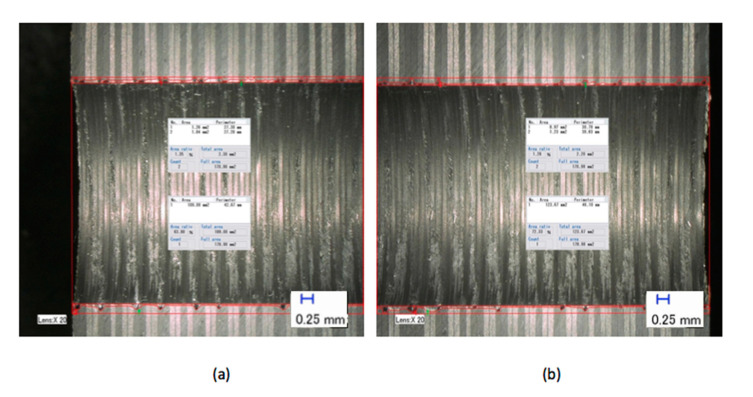
Typical example of cross-section measurement for the extension of adjusted delamination factor (S_FDSR_) at a feed rate of 0.3 mm/rev and a cutting speed of 64 m/min: (**a**) bottom part; (**b**) top part.

**Figure 8:**
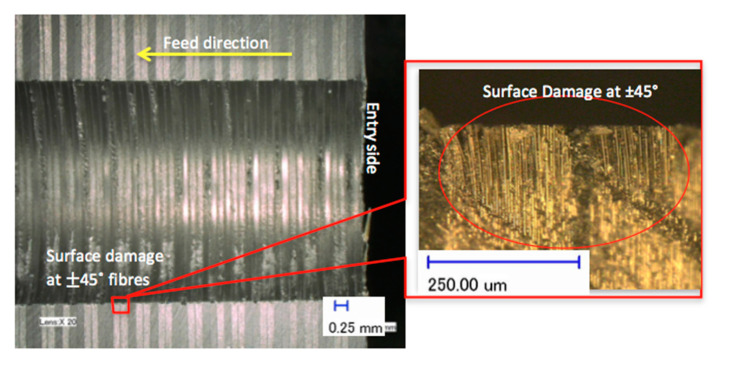
Drilling effect when the fibres were normal to the cutting direction.

**Figure 9:**
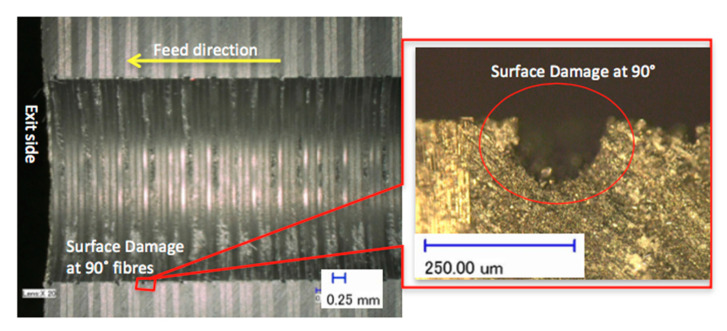
Drilling effect when the fibres were running parallel to the cutting direction.

The features of the S_FDSR_ approach were observed at the entry sides of the drilled samples only. This was because most of the results achieved a value of more than 1.101 (see Figure 5 and Figure 6), and it was decided to proceed with this approach after obtaining the results of F_da_ at hole entry due to the high severity of damage compared with the results at the hole exit. Furthermore, with the S_FDSR_ approach, it is not necessary to compute values at both positions. It is recommended that the potential user should identify which one is the most severe, i.e., hole entry or exit. The purpose of choosing one side is to avoid a repetition of calculating the damage. Since one side has already obtained significant damage, this can be used to represent the damage adjacent to the overall cylindrical shape. Before computing the S_FDSR_, a sample must be cut into cross-section; then, a digital optical microscope is used to measure the damage zone, which occurs at the edge spots. Based on the findings, the peel-up delamination increased significantly as the feed rate increased. The use of a constant feed rate with an increased spindle speed, on the other hand, appeared to minimise peel-up delamination. The action took place as the upper lamina was separated by a peeling force in the upward position from the uncut portion, which was still held by the downward-acting thrust force. The essence of this scenario is the optimisation of the peeling force, which plays an intermediate role. In practice, the thrust force and torque were the components for the peeling force, and this was proven in the recent experiments by Sobri [19]. This is significant because they are linked to the onset of delamination. Figure 7 shows a typical example of how to measure the area corresponding to A_MAXdcs_, with the assistance of image digitalisation and processing systems. It can be seen that the removal of these layers after drilling caused damage around the drilled hole and left small gaps inside the hole. Moreover, there is a series of missing lay-up sequence plies at the edge of the cross-section. It is believed that the interaction between fibre direction and the direction of cutting velocity significantly affected the machined surfaces. The surface was destroyed, and cracks were formed that penetrated into the composite during machining at 45° and 90° fibre orientations up to a length of 0.2 m—see Figure 8 and Figure 9. However, no serious damage occurred in other fibre orientations. Although there were a few cracks in the composite, the surface was smoother. The damage became worse when the cutting edges reached the bottom part of the hole, possibly caused by sensitivity to deformation due to decreased thickness. A similar trend also occurred for various combinations of cutting speed and feed rate.

**Figure 10:**
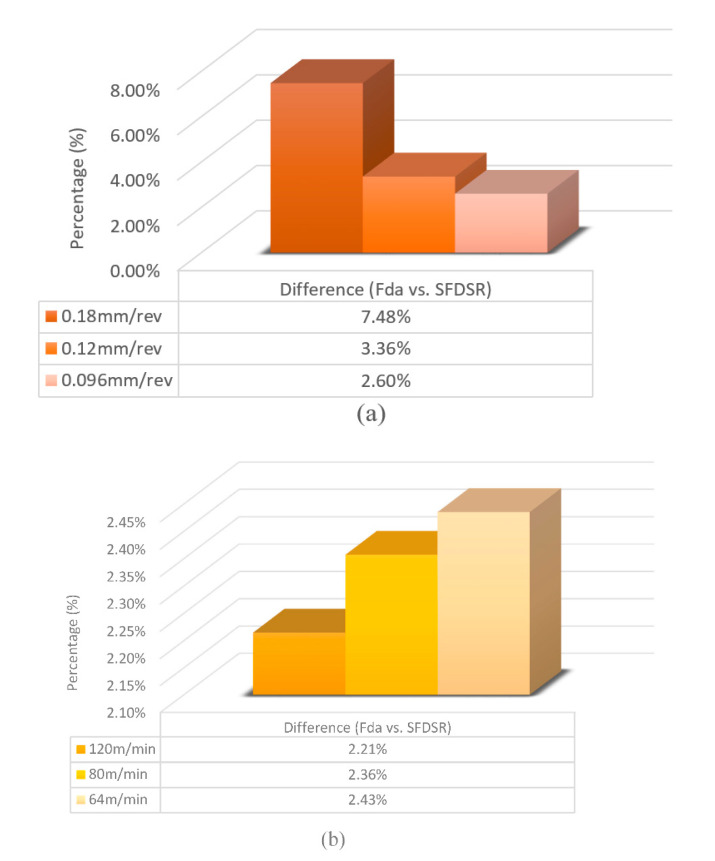
Difference (%) between both the delamination factors at: (**a**) a constant cutting speed; (**b**) a constant feed rate. Difference (%) = (S_FDSR_ − F_da_/ S_FDSR_) × 100.

After measuring the damage in the cross-section area, all relevant values were included in the S_FDSR_ equation in order to observe the interaction effects of cutting speed and feed rate. Based on the delamination factor results, it can be noted that the damage area increases due to the susceptibility of higher feed variations. Alternatively, in contrast to cutting speeds, minimum delamination can be achieved by increasing the feed rate, which highlights the significance of drilling thick CFRP. The delamination level reached or exceeded the threshold of 1.201 and potentially compromised the structural integrity and hole appearance. The samples exceeded the threshold of 1.301, which indicates extremely inferior structural integrity and hole appearance. Figure 5 shows the examples of delamination and fibre overhangs. This could be caused by the softening of the matrix, which allowed the fibres to deflect and not to be cut entirely by the tools during the drilling operation. Furthermore, drilling heat generation softened the matrix, which can influence its shear modulus and mitigate the radial stresses of the fibres. The differences between both approaches (F_da_ and S_FDSR_) can be clearly seen by comparing them in Figure 10, and it is agreed that S_FDSR_ allows an improved version of visualisation of the variations in the damage extension after drilling thick CFRP. The most significant factor affecting delamination is feed rate variations, which is in line with the results of other researchers. In addition, based on the series of missing lay-up sequence plies at the edge of the cross-section area, this modified version of the delamination factor may be used in the future to develop an interaction relationship between cutting edge and thickness/fibre orientation of the material. S_FDSR_ has been achieved at the entrance of the hole with a peak ratio of 1.39 at a cutting speed and feed rate of 64 m/min and 0.18 mm/rev, respectively, which are undesirable (i.e., third level). At a cutting speed and feed rate of 120 m/min and 0.096 mm/rev, respectively, the lowest S_FDSR_ ratio of 1.174 is the optimal performance. The speed–feed combinations should not, based on those figures, be extended beyond the S_FDSR_ ratio (i.e., 1.174) to avoid major post-processing and to ensure that the internal structure of the hole is in good condition. Delamination at the hole entry is highly dependent on the feed rate, as the increase in the feed rate resulted in an increase in the delamination factor. Nevertheless, it should be noted that none of the speed–feed combinations resulted in the first level of the delamination factor.

## 5. Conclusions

The research work presented in this paper is concerned with the drilling of carbon fibre-reinforced polymer composites (CFRP), and an experimental study of drilling holes in approximately 25.4 mm thick CFRP was conducted. A number of conclusions can be drawn based on the results and discussion, which can be referred to for future enhancement of the delamination factor in drilling of CFRP:

- The extension of the adjusted delamination factor (S_FDSR_) was able to define the damage done by drilled thick CFRP composites, which explicitly demonstrated the damage intensity inside the hole as the drilling parameters were altered.
- Taking into account the damage region of the delamination factor at the opening of the hole, the representation of the damage extension variations after the drilling of thick CFRP composites was enhanced by analysing the internal damage, i.e., inside the hole. With the aid of image digitisation and processing systems, the region corresponding to A_MAXdcs_ shows that the removal of these layers after drilling created damage around the drilled hole and left minor gaps within the hole.
- A standard guideline for the delamination factor ratio has been proposed, and every industry may refer to this guideline for the quantification of damage. Three levels of the delamination factor ratio have been established. The first level is appropriate hole consistency with a minimum delamination of between 1.000 and 1.100. The second level is acceptable; a post-machine phase may be required, but a minimum amount of rework is required, varying from 1.101 to 1.200. Lastly, the third stage is extreme and inappropriate, with a ratio of more than or equal to 1.201.

## Figures and Tables

**Table 1:** Properties of carbon fibre-reinforced polymer composites (CFRP).

Material	Grade	Yield Strength	Density
CFRP	M21	835 MPa	2.06 g/cm^3^

## References

[1] Lau W.S., Yue T.M., Lee T.C., Lee W.B. (1995). Un-conventional machining of composite materials. J. Mater. Process. Technol..

[2] Shanmugam D.K., Chen F.L., Siores E., Brandt M. (2002). Comparative study of jetting machining technologies over laser machining technology for cutting composite materials. Compos. Struct..

[3] Hernandez-Castaneda J.C., Sezer H.K., Li L. (2011). The effect of moisture content in fibre laser cutting of pine wood. Opt. Lasers Eng..

[4] Santhanakrishnan G., Krishnamurthy R., Malhotra S.K. (1988). Machinability characteristics of fibre reinforced plastics composites. J. Mech. Work. Technol..

[5] Garrick R. Drilling Advanced Aircraft Structures with PCD (Poly-Crystalline Diamond) Drills. https://www.sae.org/publications/technical-papers/content/2007-01-3893/.

[6] Mangalgiri P.D. (1999). Composite materials for aerospace applications. Bull. Mater. Sci..

[7] Reza N. (2010). Laser Cutting of Carbon Fibre-Reinforced Polymer Composite Materials. Ph.D. Thesis.

[8] Davim J.P., Rubio J.C., Abrao A.M. (2007). A novel approach based on digital image analysis to evaluate the delamination factor after drilling composite laminates. Compos. Sci. Technol..

[9] Faraz A., Biermann D., Weinert K. (2009). Cutting edge rounding: An innovative tool wear criterion in drilling CFRP composite laminates. Int. J. Mach. Tools Manuf..

[10] Shyha I.S., Aspinwall D.K., Loo S.L., Bradley S. (2009). Drill geometry and operating effects when cutting small diameter holes in CFRP. Int. J. Mach. Tools Manuf..

[11] Shyha I.S., Soo S.L., Aspinwall D., Bradley S. (2010). Effect of laminate configuration and feed rate on cutting performance when drilling holes in carbon fibre reinforced plastic composites. J. Mater. Process. Technol..

[12] Ramulu M., Branson T., Kim D. (2001). A study on the drilling of composite and titanium stacks. Compos. Struct..

[13] Sheikh-Ahmad J.Y. (2009). Machining of Polymer Composites.

[14] Chen W.C. (1997). Some experimental investigation in the drilling of carbon fiber reinforced plastic (CFRP) composite laminates. Int. J. Mach. Tools Manuf..

[15] Aoyama E., Hiromich N., Toshiki H. (2001). Drilled hole damage of small diameter drilling in printed wiring board. J. Mater. Process. Technol..

[16] Sobri S.A., Heinemann R., Whitehead D., Shuaib N. (2018). Drilling Strategy for Thick Carbon Fiber Reinforced Polymer Composites (CFRP): A Preliminary Assessment. J. Eng. Technol. Sci..

[17] Nardone F., Lignola G.P., Prota A., Manfredi G., Nanni A. (2011). Modeling of flexural behavior of RC beams strengthened with mechanically fastened FRP strips. Compos. Struct..

[18] Machining Carbon Fibre Materials. http://www.sandvik.coromant.com/sitecollectiondocuments/downloads/global/technical%20guides/en-gb/c-2920-30.pdf.

[19] Sobri S.A. (2018). Mechanical and Laser Drilling of Thick Carbon Fibre Reinforced Polymer Composites (CFRP). Ph.D. Thesis.

